# The intersection of heart failure and cancer in women: a review

**DOI:** 10.3389/fcvm.2024.1276141

**Published:** 2024-02-28

**Authors:** Sara Tyebally, Ching-Hui Sia, Daniel Chen, Aderonke Abiodun, Mayank Dalakoti, Po Fun Chan, Chieh-Yang Koo, Li Ling Tan

**Affiliations:** ^1^ Division of Cardiology, Department of Medicine, Ng Teng Fong General Hospital, Singapore, Singapore; ^2^ Institute of Cardiovascular Science, University College London, London, United Kingdom; ^3^ Department of Cardiology, National University Heart Centre Singapore, Singapore, Singapore; ^4^ Department of Medicine, Yong Loo Lin School of Medicine, National University of Singapore, Singapore, Singapore; ^5^ Hatter Cardiovascular Institute, University College London, London, United Kingdom; ^6^ Department of Cardiology, Princes of Wales Hospital, Sydney, NSW, Australia; ^7^ Department of Cardiology, NUS Cardiovascular Metabolic Disease Translational Research Program, Singapore, Singapore

**Keywords:** cancer, heart failure, women, prevention, cardio-oncology

## Abstract

Cancer and cardiovascular disease represent the two leading causes of morbidity and mortality worldwide. Women continue to enjoy a greater life expectancy than men. However, this comes at a cost with more women developing diabetes, hypertension and coronary artery disease as they age. These traditional cardiovascular risk factors not only increase their lifetime risk of heart failure but also their overall risk of cancer. In addition to this, many of the cancers with female preponderance are treated with potentially cardiotoxic therapies, adding to their increased risk of developing heart failure. As a result, we are faced with a higher risk population, potentially suffering from both cancer and heart failure simultaneously. This is of particular concern given the coexistence of heart failure and cancer can confer a worse prognosis than either a single diagnosis of heart failure or cancer alone. This review article explores the intersection of heart failure and cancer in women at multiple levels, including traditional cardiovascular risk factors, cardiovascular toxicity derived from antineoplastic and radiation therapy, shared pathophysiology and HF as an oncogenic process. This article further identifies opportunities and strategies for intervention and optimisation, whilst highlighting the need for contemporary guidelines to better inform clinical practice.

## Introduction

The prevalence of heart failure (HF) and cancer have both increased alongside an ageing population (1, 2) and can lead to significant morbidity and mortality in women. Recent studies have demonstrated that HF and cancer often coincide given the complex interplay in shared clinical and social risk factors such as diabetes mellitus, hypertension, dyslipidaemia, smoking and obesity (3, 4). Multiple studies have already demonstrated that women with cancer are more likely to be comorbid and suffer from pre-existing cardiovascular risk factors (5–8). Moreover, several antineoplastic therapies such as anthracyclines and HER2-targeted therapies (used more commonly in females to treat breast cancer), and radiation therapy are potentially cardiotoxic, adding to the likelihood of developing HF. In addition to the above, inflammation and oxidative stress, age and genetic predisposition, and HF as an oncogenic process in itself may potentiate both HF and cancer in women (4).

As a consequence, the burden of HF amongst female patients with cancer is expected to rise, and this is of concern given that the co-existence of HF and cancer can confer a worse prognosis than either a single diagnosis of HF or cancer alone (9–12), and unfortunately this risk is higher in women than men (12).

As such, opportunities to screen and manage cardiovascular risk factors in women with cancer is paramount, with the goal of developing targeted prevention strategies to potentially minimise the likelihood of progression to HF in this population. Unfortunately, traditional cardiovascular risk scores do not take into account additional cardiovascular risks incurred from cancer and its therapies.

This review article will explore the complex and bidirectional nature of the interaction between cancer and HF in women, whilst identifying potential opportunities and strategies for intervention and optimisation.

## Intersection of heart failure and cancer

**Figure 1:**
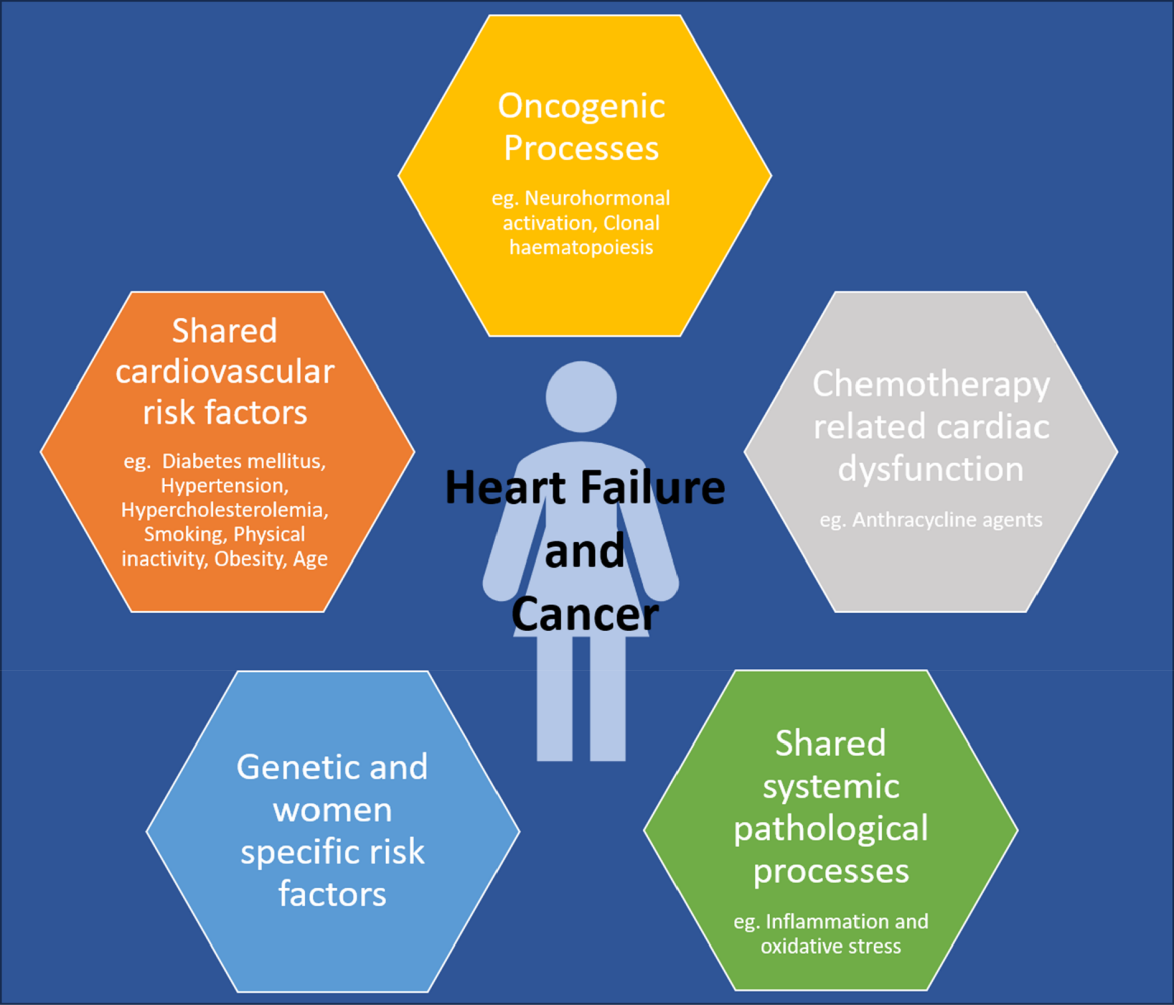
Depicting the intersection of heart failure and cancer in women.

It has been established that HF and cancer may accompany each other, given its complex intersection at multiple levels as described above. Discussed below and depicted in Figure 1 are potential pathways linking HF and cancer in women.

## Shared traditional cardiovascular risk factors

### Diabetes mellitus

The relationship between diabetes and HF is well described in the literature. Termed “diabetic cardiomyopathy”, this is referred to as a process that affects cardiac function and structure independent of cardiovascular risk factors and age, or events which can lead to systolic or diastolic HF (13). In females, a recent study has shown that women with diabetes (Type I and Type II) experience an up to 22% increased risk of HF compared to male counterparts [HR of 2.2 (95% CI: 1.9–2.5) vs. 1.8 (1.7–2.0) respectively], and that this increased risk was independent of confounding factors (14).

An increased incidence of cancer is also observed in patients with diabetes. Several proposed mechanisms include increased oxidative stress, increased inflammation, and direct effects of excess insulin and glucose signalling (15, 16). In addition, many chemotherapeutic regimens include glucocorticoids which may exacerbate pre-existing diabetes or further induce diabetes (17).

Overall, it is thought that almost 20% of women with cancer have diabetes (18), highest in females with pancreatic cancer (up to 37%) (18). Cancer patients with concomitant diabetes are known to experience a significantly poorer prognosis than those without diabetes (19, 20), which may then impact treatment practices and affect cancer survivorship.

### Hypertension

Hypertension is also a well-established risk factor for HF. However despite similar prevalence of hypertension in women and men, the risk of developing HF is greater in hypertensive women when compared to their male counterparts (2-fold in men and 3-fold in women) (21).

The prevalence of hypertension is also greater in cancer patients when compared with the general population. In fact, it is the most common modifiable risk factor of adverse cardiovascular outcomes amongst cancer patients (22). Less is known about the association of blood pressure (BP) with cancer; however chronic inflammation has been proposed as a potential mechanism (23). In addition, experimental studies have demonstrated a potential role of the renin-angiotensin-aldosterone system, crucial in the regulation of BP, in the biological processes of angiogenesis, inflammation, cellular proliferation, and tissue remodelling (24).

Several cancer treatments such as vascular endothelial growth factor inhibitors (VEGFi) are also associated with the exacerbation or development of hypertension (25).

In cancers specific to women, a meta-analysis performed by Han et al. demonstrated a positive association between hypertension and breast cancer risk. Women with hypertension may have a 15% increased risk of breast cancer but this was only found in postmenopausal women and not in premenopausal women (26). Large European case–control and cohort studies have consistently demonstrated a higher risk of cancer with high BP [odds ratio were 1.2 (95% CI: 1.1–1.4) for breast cancer and 1.6 (95% CI: 1.3–1.9) for endometrial cancer, and these increased odd ratios persisted after 5 years or more since the diagnosis of hypertension] (27–29).

This is of concern as a large prospective cohort study demonstrated that elevated BP levels were significantly associated with increased cancer mortality in women (30). Cancer risk was also found to correlate linearly with increasing BP levels; however, for both cancer mortality and incidence, this association was more profound in men than women [hazard ratios per 10 mmHg BP increment of 1.06 (95% CI: 1.02–1.11) for women and 1.12 (95% CI: 1.08–1.15) for men] (30).

### Hypercholesterolaemia

Hypercholesterolaemia is a pertinent and independent risk factor in the development of ischaemic heart disease, and therefore potentially leading to HF. Whilst elevated levels of total cholesterol have not demonstrated to be a strong predictor of new-onset HF (31, 32), an increased ratio of total cholesterol to HDL cholesterol is associated with increased HF risk (33).

Unfortunately, the relationship between hypercholesterolaemia and cancer has not been well established. However, it has been hypothesised that the chronic intake of saturated fats and cholesterol may lead to an increase in hepatic bile acids which may subsequently promote carcinogenesis (34, 35). In one experimental study, it was postulated that hypercholesterolaemia could accelerate and enhance tumour formation in females with breast cancer (36). In addition, these tumours were found to be more aggressive, with tumour angiogenesis enhanced (37).

### Age

Age remains the single strongest predictor of adverse cardiovascular events. The occurrence of HF is elevated with advancing age, and women of an older age are at greater risk than men (38). Alongside, CVRFs also increase with advancing age with its incidence reported to be as high as 78.2% in females aged 60–79 years and increasing to over 90% in females above the age of 80 years (39). Women tend to develop HF at an older age when compared to men, and more commonly have HFpEF or HF with mildly reduced ejection fraction [LV ejection fraction (LVEF) 41%–49%] than when compared to men (40).

The incidence of most cancers also increases with advancing age and in developed countries, almost 78% of all newly diagnosed cancers occur in individuals over the age of 55 years (41). Among people aged over 65, incidence rates are around 50% higher in men than in women. It is however important to note that amongst individuals aged 25–59, the incidence rates are higher in women than in men (42).

### Obesity

The prevalence of obesity has increased at a significant rate in almost all developed and developing countries, reaching alarming levels of up to 60%–70% of the adult population in industrialised countries (43). It has been established that the relationship between obesity and HF is complex given multiple shared variables that may interact such as age, diabetes, hypertension, and even educational level (44). The global prevalence of obesity is higher in women, with the risk of developing HFpEF increasing by up to 34% for every standard deviation increase in body mass index (45). Although women with HFpEF are around 20% less likely to experience hospitalisation or death when compared to men (46), this survival appears to be counteracted by a decrease in their quality of life, as women living with HF are more likely to self-report higher physical disability and psychological scores (47).

Worldwide, the burden of cancer attributable to obesity is 13.1% in women (48). Proposed mechanisms include a chronic state of low-level inflammation, potentially leading to DNA damage, thus increasing the likelihood of cancer mutations and incidences (49).

Interestingly, there has been recent epidemiological data supporting the hypothesis that obesity may be protective against certain types of cancer, pertaining to their mortality and incidence. For example, obesity is associated with a decreased risk of breast cancer in premenopausal women by about 10% (50), and in endometrial cancer, good prognosis type 1 tumours features more commonly when compared to poor prognosis type 2 tumours. Potential hypotheses of the obesity paradox in patients with cancer may include methodologic issues such as body mass index as an inadequate measure of adiposity, confounding variables and reverse causality. Clinical variables may include less aggressiveness of tumour biology in obesity, and nutritional reserve as a mean of patient selection for anti-cancer treatments (51, 52).

### Smoking

Smoking is a notable modifiable risk factor in both HF and cancer. Cigarette smoking has been found to be independently associated with an increased risk of HF in women than men; 88% in women compared to 45% in men (53).

A recent study by Lai et al. demonstrated that a significantly increased risk of overall [1.25-fold higher (95% CI: 1.08–1.45; *P* = 0.0022)] and cancer specific [1.22-fold higher (95% CI: 1.04–1.44; *P* = 0.0168)] mortality was found in breast cancer women who were current smokers when compared to their non-smoker counterparts. Among these women, ex-smokers had a lower mortality risk than current smokers [1.57-fold higher risk (95% CI: 1.02–2.42; *P* = 0.0407)] (54).

### Physical activity

Data on the association between physical activity and HF have demonstrated that physical activity may confer a protective benefit in reducing the risk of developing subsequent HF (55). Berry et al. found that low fitness remained strongly associated with HF risk (14.3% in the low fitness cohort vs. 4.2% in the high fitness cohort) (56).

Exercise has also been linked to not only a decrease in the primary incidence of cancer but also the recurrence in cancer survivors. It has been demonstrated that sedentary women who embarked on an exercise regimen of 150 min weekly were found to have decreased their risk of breast cancer recurrence by about 6% (57). Cancer preventive effects were found to be independent of body mass; an inactive individual with a healthy body mass in effect has a higher risk for cancer when compared to in those who engage in regular exercise (58).

### Coronary artery disease

All the aforementioned risk factors increase the risk of atherosclerosis and may eventually lead to myocardial infarction, which remains the most important risk factor for incident HF. Women are 2–3 times less likely to have coronary artery disease (CAD) compared to men; however when they do develop CAD, they are more likely to develop HF (59). Cancer and CAD also share common biological pathways with inflammation being a major unifying factor in the aetiology and progression of these diseases (60). Studies have shown that women with a history of prior myocardial infarction are more likely to develop cancer (61), and the risk of cancer is even higher in patients who subsequently develop HF after a myocardial infarction (62).

## Cancer treatment related cardiac dysfunction CTRCD

While cancer-free survival has increased, complications from cancer therapies, particularly a deterioration in cardiac function (Table 1), have limited patient outcomes and impacted the overall mortality and morbidity adversely (63).

Anticancer therapies can cause HF directly through their cardiotoxic effects or indirectly through other mechanisms such as ischaemia, myocarditis, systemic or pulmonary hypertension, valvular heart disease or arrhythmias. Molecular targeted therapies, cytotoxic chemotherapy and mediastinal radiotherapy have all been linked to HF and can result in a 3.5-fold increased mortality risk compared to patients with idiopathic cardiomyopathies (64).

Identification of patients at risk of CTRCD is difficult though very important. Patient-related risk factors include pre-existing cardiovascular risk factors as mentioned above (65). In addition to this, gender differences may also play a significant role where susceptibility to CRTCD is concerned. Sex specific differences in pharmacodynamics and pharmacokinetics (absorption, distribution, excretion) may have important clinical consequences. For example, several authors have showed that men have a significantly higher anthracycline clearance than women, which could be related to greater aldoketoreductase activity (66, 67), lowering their likelihood of developing cardiotoxicity. Moreover, reduced expression of p-glycoprotein found in females may lead to high rates of cardiotoxicity secondary to anthracyclines (68). On the other hand, female sex hormones may be protective against oxidative stress and mitochondrial dysfunction caused by anthracyclines, which have been found to be a contributory factor towards anthracycline cardiotoxicity (69).

Where radiotherapy is concerned, data from meta analyses have consistently shown that radiotherapy following mastectomy or breast conserving surgery may reduce the risk of local recurrence by approximately 75% (70). Unfortunately this can also result in an increased risk of cardiovascular mortality and morbidity through a spectrum of radiation-induced heart disease including accelerated coronary atherosclerosis, myocardial fibrosis, pericarditis, pericardial effusions with or without constriction, valvular heart disease and arrhythmias, all of which can eventually lead to HF (71, 72). Recent studies have also implicated radiotherapy in the development of HFpEF (73).

Unfortunately, women treated with radiation therapy may have a significantly increased odds of incident cardiovascular disease and cardiovascular mortality compared to men (OR 3.74), as found on a metaanalysis of Hodgkin's lymphoma survivors treated with radiation therapy (74).

**Table 1:** Summarises various classes of cancer therapies that are known to cause HF and their underlying mechanism of cardiotoxicity (75–83).

Cancer Therapy	Drug examples		Mechanism of Cardiotoxicity	Incidence of LVD and/or HF
Anthracycline chemotherapy	• Doxorubicin • Epirubicin • Daunorubicin • Idarubicin		Myocardial cell loss, apoptosis, and necrosis, mediated by oxidative stress	Up to 48%
HER2-targeted therapies	• Trastuzumab • Pertuzumab • Trastuzumab emtansine T-DM1 • Lapatinib • Neratinib • Tucatinib		Inhibition of HER2, impairing adaptation to stress	1.7% to 4.1%
VEGF inhibitors	TKIs	Antibodies	Depletion of coronary microvascular pericytes, inhibition of raf-1/B-raf	0.5% to 11%
	• Sunitinib • Pazopanib • Sorafenib • Axitinib	• Bevacizumab • Ramucirumab		
Multi-targeted kinase inhibitors (Second and third generation BCR-ABL TKIs)	• Ponatinib • Nilotinib • Dasatinib • Bosutinib		Inhibition of growth signalling pathways	1% to 5%
Proteasome inhibitors	• Carfilzomib • Bortezomib • Ixazomib		Apoptosis of cells due to the aberrant proteome	2% to 5%
Immunomodulatory drugs	• Lenalidomide • Pomalidomide		Direct damage of endothelial cells, increased platelet aggregation	1.7% to 1.8%
Alkylating agents	• Cyclophosphomide		Toxic effect of its metabolite on the endothelial cells	7% to 28%
Mitotic Inhibitors	• Docataxel • Paclitaxel		Impaired cell division	2.3% to 8%
Immune checkpoint inhibitors	• Nivolumab • Pembrolizumab		Immune response activation	Unknown but estimated to be around 0.5% to 1%

HER 2, human epidermal growth factor receptor 2; VEGF, vascular endothelial-derived growth factor; TKI, tyrosine kinase inhibitor.

## Genetic and women specific risk factors

The genetic background of a patient may have several implications; firstly, the susceptibility to concomitant cancer and cardiovascular disease development, secondly, the susceptibility to cancer related cardiovascular complications and lastly, the susceptibility to cardiotoxicity from antineoplastic therapies.

Genome-wide association studies have demonstrated that the most significant commonly inherited genetic variant associated with atherosclerosis resides in a well-known cancer locus situated at chromosome 9p21, rather than in a gene that regulates traditional cardiovascular risk factors (84–86).

Where CRTCD is concerned, to date, multiple genome-wide association studies have managed to identify and replicate several single nucleotide polymorphisms (SNPs) and implicated DNA damage response, sarcomere dysfunction, oxidate stress and anthracycline transport and metabolism that were linked with anthracycline induced ardiomyopathy (87, 88). Unfortunately, the effect size of the SNPs identified are weak to moderate, limiting their clinical use in individual patients.

### Women-specific risk factors

#### Pregnancy

A recent study determining the incidence of cancer in peripartum cardiomyopathy (where majority had a LVEF >50%) found women with peripartum cardiomyopathy were 9 times more likely to develop cancer following their diagnosis when compared to matched controls in Swedish and German cohorts (89). This was correlated with exome sequencing, revealing that peripartum cardiomyopathy patients had a higher prevalence of genetic variants in DNA damage repair genes and cancer promoting syndrome.

#### Menopause

Premature menopause is a common result of cancer treatment and unfortunately, is a contributing factor to cardiovascular disease in women (90). Premature menopause is associated with a 33% higher risk of developing HF, and a 24%–28% higher risk of developing cardiovascular disease, independent of traditional cardiovascular risk factors (91).

## Shared systemic pathological processes

Systemic pathological mechanisms such as inflammation and oxidative stress have considerable overlap and common final pathways linking HF and cancer. In HF, they act as powerful drivers for atherosclerosis which is the leading aetiology of HF. In addition to this, they can directly induce alterations in the myocardium, creating a substrate for the development of HF (4). Decrease nitric oxide bioavailability found in microvascular endothelial inflammation may also be lead to HFpEF, found most commonly in women (4).

In cancer, chronic inflammation with persistent cellular oxidative stress may boost cancer initiation and progression. Some inflammatory pathways are shared in HF and cancer pathogenesis. Pro-inflammatory cytokines such as interleukin (IL)-1B, IL-6, and IL-18 and tumour necrosis factor (TNF), have all been shown to play a role in left ventricular dysfunction and adverse cardiac remodelling (92). In obese women, excess interleukin-6 can result in increased cardiovascular disease risk leading to HF, whilst also inhibiting cancer cell apoptosis (93). Increased expression of these cytokines has also proven to have detrimental effects. Recently, the CANTOS (Canakinumab Anti-inflammatory Thrombosis Outcomes) trial, designed to test the hypothesis that an anti–interleukin-1ß antibody could reduce adverse cardiovascular outcomes, interestingly also found a concomitant reduction in the development of lung cancer and lung cancer mortality (94). Animal models have shown reduction in breast cancer metastasis with IL-1B inhibition (95).

## Oncogenic processes

Several preclinical studies to date have demonstrated that a weakened myocardium secretes oncogenic factors (e.g., SerpinA3, Fibronectin), contributing to tumour progression and formation (96).

In addition to this, neurohormonal activation, seen commonly in the development of cardiac remodelling and HF, has also been found to promote tumour development and progression through the activation of intracellular signalling pathways of cancer cells (12, 97). Chronic and progressive hyperactivation of the sympathetic nervous system (SNS) and RAAS is a hallmark and major component of HF, and its effects of cancer also been explored extensively; tumorigenesis can be precipitated by excess SNS activity via β-AR–dependent activation of stimulatory G protein-protein kinase A and β-arrestin-1 signalling, which then promotes the accumulation of DNA damage and impedes its repair (98). Several other pathways are also implicated; a recent animal study has demonstrated that the presence of MI-induced heart failure promoted mammary tumor cell growth through the NGF-TRKA pathways, suggesting that the inhibition of TRKA signalling pathways may be a potential therapeutic target for patients with breast cancer after MI (99). Cardiac-derived circulating factors are also thought to exert exocrine effects on tumour cells (100).

Lastly, the phenomenon of clonal haematopoiesis of indeterminate potential(CHIP) which refers to the clonal expansion of mutated myeloid cells in individuals without other hematologic abnormalities, has been found to been associated with an 11-fold higher relative risk for developing haematological malignancies and a 2- to 4-fold increased risk for developing cardiovascular disease (101). Although the precise molecular mechanism linking CHIP mutations to cardiovascular disease remains undefined, inflammasome activation has been hypothesized to be a key component to this process (102, 103).

## Investigation of heart failure in women

Although the diagnosis of HF is clinical (based on characteristic signs and symptoms), cardiac imaging with serum cardiac biomarkers help to confirm the diagnosis, understand the aetiology and facilitate patient management. Interval imaging and serial biomarkers are often required to monitor disease progression, assess treatment response and help with risk stratification.

### Serum cardiac biomarkers

Cardiac biomarkers such as B-type natriuretic peptide (BNP), N-terminal pro-B-type natriuretic peptide (NT-proBNP) and troponin are useful to support clinical evaluation, diagnosis, and prognostication of HF. In women with cancer, cardiac biomarkers may be incorporated at various phases of cancer management such as the assessment of baseline risk prior to initiating cancer treatment to guide primary prevention treatment, during treatment to monitor for early signs of cardiotoxicity, and when screening for latent side effects post cancer treatment and in cancer survivorship. Women have an increased tendency to have higher natriuretic peptide levels compared with men with decompensated HF, including those with HFrEF (median BNP in men vs. women: 1,113 vs. 1,259 pg/ml) (104). An elevated troponin in the setting of chronic HFrEF portends a poorer prognosis for both women and men, especially in the absence of significant CAD (105).

### Echocardiography

Echocardiography is generally the first line imaging modality for the assessment of LVEF [preferably with three-dimensional (3D) echocardiography] due to its wide availability, patient tolerability, lack of ionising radiation and cost effectiveness. Global longitudinal strain (GLS) has also been increasingly incorporated into guidelines as an early marker for the diagnosis of CTRCD.

To further delineate the cause of LV dysfunction, echocardiography can also assess for regional wall motional abnormalities most commonly seen in ischaemic heart disease, valvular heart disease and pericardial disease.

Unfortunately, obtaining good quality imaging in women with cancer (in particular breast cancer following mastectomy or reconstructive implant) can be challenging. The American Society of Echocardiography suggests the use of contrast echocardiography for improved endocardial border definition in patients with breast implant or prior mastectomy (106).

### Nuclear imaging

Multi-acquisition gated angiography (MUGA) has the benefit of accurately assessing LVEF. However, MUGA is not the preferred imaging modality of choice in most cases due to ionizing radiation exposure, which is of significant concern in patients with breast cancer. Other limitations include the inability to obtain other cardiac functional and structural information, which can be obtained through echocardiography (107). Physiological or pharmacological stress myocardial perfusion imaging can be used to diagnose ischaemic heart disease as a cause of HF.

### Cardiac magnetic resonance imaging

Cardiac magnetic resonance (CMR) imaging is a safe, reproducible and robust approach in the non-invasive evaluation of cardiovascular diseases particularly in women with suspected or known cardiovascular disease given the lack of ionizing radiation exposure, which can be potentially harmful to female breast tissue (108, 109) or to the foetus in pregnant patients (110).

When echocardiographic image quality is inadequate for functional and volumetric assessment, CMR can be used as the first line modality of choice (111). Furthermore, in cases where echocardiography findings are abnormal such as a change in LVEF, CMR could be performed to confirm these findings prior to making decisions about management (107). CMR is also useful in tissue characterisation and the evaluation of ischaemic vs. non ischaemic pathologies (107).

### Computed tomography coronary angiography

Computed tomography coronary angiography (CTCA) is not commonly used to assess ventricular function. However, it has high negative predictive value and excellent sensitivity in the diagnosis of CAD, particularly in patients who have received radiotherapy in the past. Where cancer patients often may have clotting or platelet abnormalities secondary to their cancer or cancer treatment henceforth making them high-risk candidates for invasive coronary angiography, CTCA may be utilised as a lower risk alternative with studies showing high correlation with angiographic findings (112).

## Management of heart failure in women

Currently, there are no sex-specific HF guidelines because women traditionally have been underrepresented in clinical trials (average female participation is 20%) and sex-specific data are rarely analysed prospectively (113). Amongst the established medical therapies for HFrEF, angiotensin-converting enzyme inhibitors (ACEIs), angiotensin receptor blockers (ARBs), beta blockers, mineralocorticoid receptor antagonists (MRAs),ivabradine and newer agents such as angiotensin receptor-neprilysin inhibitor (ARNI) and sodium-glucose co-transporter 2 inhibitors (SGLT2i) have been shown in randomised controlled trials to improve symptoms, reduce HF hospitalisation, and decrease mortality (114). Uptitration of HF therapies to the maximum tolerated dosage is a key element in managing HF patients in order to improve patient outcomes. Unfortunately, surveys have shown that women with HF are less likely to receive treatment with ACEIs, beta blockers and MRAs compared with men (115, 116). Initiating first line therapy in HF may also prove challenging in cancer patients who often have a higher likelihood of chronic kidney disease.

For patients with HFpEF, an essential component of treatment would be managing contributing factors and frequently present comorbidities such as obesity as this can significantly impact the clinical course.

### Angiotensin-converting enzyme inhibitors

ACEIs are currently recommended in all HF patients with impaired systolic function due to their known morbidity and mortality benefits(in the absence of any contraindications) (117). A large meta-analysis consisting of 30 ACEI studies in women with heart failure (*N* = 1,587) showed a trend towards improved survival and symptom reduction in the arm taking ACEIs compared with those who did not (118, 119). Several studies also demonstrated the protective effects of ACEIs in chemotherapy-induced LV dysfunction in women with cancer (120).

### Angiotensin receptor blockers

ARBs are typically used in ACEI-intolerant patients. Sex-specific data for ARBs are limited, but candesartan (121) and valsartan (122) seem beneficial in women, having been shown to reduce the combined endpoint of cardiovascular death and HF hospitalisations in women.

### Beta blockers

Bisoprolol, carvedilol and metoprolol succinate, when taken in tandem with ACEI, are beneficial in women with HF despite the relatively small number of female participants in each study (123–126). In women with breast cancer, the initiation of prophylactic carvedilol or nebivolol prior to anthracycline-based chemotherapy has been shown to be beneficial in small randomised, placebo-control trials, demonstrating less reduction in LV function at six months than placebo (127, 128).

### Mineralocorticoid receptor antagonists

MRAs are one of the few therapeutic interventions deemed by subgroup *post hoc* analysis, to have a total mortality benefit for women with systolic HF (129, 130). In women with cancer, spironolactone administered simultaneously along with anthracycline resulted in a reduced deterioration in ejection fraction and diastolic stabilisation (131).

### Ivabradine

Ivabradine has proven to demonstrate a significant reduction in hospitalisation for worsening HF, with similar effectiveness found in women and men from subgroup analysis (132).

### Angiotensin receptor-neprilysin inhibitors

Women were observed to derive more benefit from sacubitril/valsartan compared with men, an effect mainly driven by reductions in HF hospitalisations (133). In women with HFpEF, sacubitril/valsartan did not result in a significantly lower rate of HF hospitalisations and death (134).

### Sodium-glucose co-transporter 2 inhibitors

Meta-analyses have demonstrated that SGLT2i lower the risk of cardiovascular death, all-cause mortality, and hospitalisation for HF (1–3, 5) in patients with underlying HF regardless of their LVEF, with the benefits more pronounced in women (135). A recent study demonstrated that SGLT2 inhibitors were also associated with lower rate of cardiac events among patients with cancer and DM in patients who were treated with anthracyclines, however, randomized trials testing the effect of SGLT2 inhibitors on cardiac outcomes in patients treated with anthracyclines are still required (136).

### Metformin

There might be a potential role in the use of metformin to prevent HF in women receiving anthracycline therapy. A recent study found that the use of metformin was associated with a lower incidence of HF (cumulative incidence: 3.6% vs. 10.5%; *P* = 0.022; HR: 0.35; 95% CI: 0.14–0.90; *P* = 0.029) and overall mortality (HR: 0.71; 95% CI: 0.50–1.00; *P* = 0.049) in patients with DM receiving anthracyclines (137). Recently Serageldin et al. conducted a randomised study of 70 women without DM who were treated with anthracyclines for breast cancer in the adjuvant setting, and were randomised to receive metformin or control. They had found that the left ventricular ejection fraction at treatment end was minimally numerically higher in the metformin arm, although statistically significant (65.9% vs. 62.2%; *P *= 0.0007) (138).

### Devices

Examples of devices include implantable cardioverter defibrillator (ICD) and cardiac resynchronisation therapy (CRT). Patients with ischaemic cardiomyopathy tend to have less improvement in LV function due to myocardial scar tissue which has a decreased tendency to undergo favourable remodelling (139). Despite women being more likely than men to respond to CRT possibly due to smaller body and heart sizes (140, 141), recent data have shown that women are less likely to receive a device (142), and even when they do, they suffer an increased rate of implantation-related complications such infection and pneumothorax (143).

In women with cancer, specifically breast cancer, two broad challenges persist pertaining to devices and receiving anti-cancer treatment in the form of radiotherapy; firstly, device malfunction from radiotherapy and secondly, suboptimal delivery of radiotherapy due to the device overlaying the area of interest. As a result, an estimated 0.8% of all radiotherapy patients will require a modification in their radiotherapy approach because of their cardiac devices (144).

### Cardiac rehabilitation and lifestyle modifications

There is strong evidence, as reflected in the guidelines, for cardiac rehabilitation (including education), and lifestyle modifications in improving outcomes in HF patients (145). Lifestyle modifications include salt reduction, smoking cessation, weight loss, and exercise. This is particularly helpful in women with HFpEF with studies demonstrating cumulative and significant positive effects of the combination of aerobic exercise training and caloric restriction among obese older patients with HFpEF (146).

In addition, regular physical exercise is found to be an effective tool for reducing stress, depression and fatigue, and improving physical fitness and quality of life in breast cancer patients (147, 148). Unfortunately, this may prove a struggle in a certain cohort of female patients with cancer who suffer from cancer-related fatigue, limiting them to passive activities only (149).

## Management of traditional cardiovascular risk factors

Strategies aimed at optimisation of traditional cardiovascular risk factors are essential to reduce the likelihood of progression to HF in women. Controlling cardiovascular risk factors in women with cancer is also beneficial given that these risk factors are associated with an increased risk of developing CTRCD.

Generally, treatment of hypertension with antihypertensive agents is particularly effective in preventing HF. However, patients with cancer have traditionally been excluded in large-scale hypertension trials. Importantly, the treatment of hypertension should not compromise the outcome of cancer treatment. Where chemotherapy-induced hypertension is suspected, no studies have compared the efficacy of different antihypertensive agents in treating this condition. Therefore, in essential hypertension or chemotherapy-induced hypertension, ACEIs, ARBs and dihydropyridine calcium channel blockers are all considered viable first line therapies (150).

Adequate control of hypercholesterolaemia with statin therapy is also paramount in reducing the likelihood of CAD and subsequent HF (151). In addition to their lipid-lowering effect, statins are also known to have pleiotropic anti-inflammatory effects that theoretically may attenuate CTRCD. Abdel-Qadir et al. demonstrated that women on statins had a significantly lower risk of HF hospitalisation after receiving anthracycline-based chemotherapy for early breast cancer, and potentially lower risk in those receiving HER2 targeted therapy as well (152).

With regards to the management of diabetes in cancer patients, there is a paucity of data from randomised controlled trials to guide glycaemic targets. Hence a pragmatic approach to glycaemic control should be undertaken to ensure that the patient is kept asymptomatic with low risk of acute decompensation. Observational evidence, however, suggests that poor glycaemic control may contribute to poorer outcomes in cancer patients (153), as this may be due to increased risk of infections and hospitalisations whilst undergoing treatment (154, 155). There has also been data suggesting that metformin may actually reduce cancer risk (156, 157). A study by Tseng found that in comparison to non-diabetic women, the risk of developing ER-positive breast cancer was reduced by up to 38% amongst women with type 2 diabetes who had used metformin for over a decade or more (158).

The management of CAD in women with cancer may pose several challenges because they are often excluded from prospective trials assessing the efficacy and safety of acute coronary syndrome treatment. However, management of CAD in cancer patients typically follows the 3 standard possibilities as the general population: medical management, percutaneous intervention or coronary artery bypass surgery. The need for personalised care is of high importance. Key considerations that will influence decision making in the management of CAD include the stage and prognosis of the malignancy, severity of cardiac disease, comorbidities and lastly premorbid function of the patient. Potential concerns that may arise when deciding antiplatelet therapy or performing invasive procedures include the presence of anaemia, thrombocytopenia and coagulopathy in cancer patients (159).

## Conclusion

Management of shared risk factors for HF and cancer must be in tandem, through greater integration and coordination of care between the oncologist, cardio-oncologist, and primary care provider. These include early detection and better assessment and management of baseline cardiovascular risk factors, and improved monitoring of at-risk patients. Unfortunately, in women with cancer, there is limited data determining the extent to which these cardiovascular risk targets should be pursued. While it is well-established that tight control of cardiovascular risk factors will reduce the likelihood of HF development in the non-cancer population, little is known whether this practice benefits cancer patients.

In addition to this, knowledge on the impact of genetic factors on the risk of cardiotoxicity may also provide several therapeutic potentials moving forward, such as a more personalised approach to the selection of antineoplastic therapies based on cardiotoxicity profiles as well as the identification of potentially new targets for cardiotoxicity prevention in patients. Genetic testing may also play a crucial role in the future, however again, further studies focusing on integrating genetic variants or polygenic risk scores with patient and treatment related risk factors are required to facilitate and improve individual prediction and risk stratification for heart failure in cancer patients and survivors. Further advancements in inflammation research in both cardiovascular disease and cancer would also greatly benefit and refine therapeutics in this area that are safe, efficacious and cost-effective.

Most importantly, more research and greater participation of women in clinical trials are needed. Improved risk assessments and the development of personalised preventive strategies in cancer survivorship programs are imperative for improved outcomes and reductions in cardiovascular mortality in women with cancer.
